# Academic institution extensive, building-by-building wastewater-based surveillance platform for SARS-CoV-2 monitoring, clinical data correlation, and potential national proxy

**DOI:** 10.1371/journal.pgph.0003756

**Published:** 2025-05-09

**Authors:** Arnoldo Armenta-Castro, Mariel Araceli Oyervides-Muñoz, Alberto Aguayo-Acosta, Sofia Liliana Lucero-Saucedo, Alejandro Robles-Zamora, Kassandra O. Rodriguez-Aguillón, Antonio Ovalle-Carcaño, Roberto Parra-Saldívar, Juan Eduardo Sosa-Hernández

**Affiliations:** 1 Tecnologico de Monterrey, School of Engineering and Sciences, Monterrey, Mexico; 2 Tecnologico de Monterrey, Institute of Advanced Materials for Sustainable Manufacturing, Monterrey, Mexico; 3 Magan Centre of Applied Mycology, Cranfield University, Cranfield, United Kingdom; University of Oxford, UNITED KINGDOM OF GREAT BRITAIN AND NORTHERN IRELAND

## Abstract

In this work, we report on the performance of an extensive, building-by-building wastewater surveillance platform deployed across 38 locations of the largest private university system in Mexico, spanning 19 of the 32 states, to detect SARS-CoV-2 genetic materials during the COVID-19 pandemic. Sampling took place weekly from January 2021 and June 2022. Data from 343 sampling sites was clustered by campus and by state and evaluated through its correlation with the seven-day average of daily new COVID-19 cases in each cluster. Statistically significant linear correlations (p-values below 0.05) were found in 25 of the 38 campuses and 13 of the 19 states. Moreover, to evaluate the effectiveness of epidemiologic containment measures taken by the institution across 2021 and the potential of university campuses as representative sampling points for surveillance in future public health emergencies in the Monterrey Metropolitan Area, correlation between new COVID-19 cases and viral loads in weekly wastewater samples was found to be stronger in Dulces Nombres, the largest wastewater treatment plant in the city (Pearson coefficient: 0.6456, p-value: 6.36710^−8^), than in the largest university campus in the study (Pearson coefficient: 0.4860, p-value: 8.288x10^−5^). However, when comparing the data after urban mobility returned to pre-pandemic levels, correlation levels in both locations became comparable (0.894 for the university campus and 0.865 for Dulces Nombres). This work provides a basic framework for the implementation and analysis of similar decentralized surveillance platforms to address future sanitary emergencies, allowing for an efficient return to priority in-person activities while preventing university campuses from becoming transmission hotspots.

## 1. Introduction

In recent years, wastewater-based surveillance (WBS) has emerged as a powerful toolset to provide data regarding public and environmental health status in communities through the detection and quantification of specific biomarkers in the sewage system [1,2]. While initial studies focused on chemical indicators of exposure to drugs, pharmaceuticals, contaminants, and personal care products, among others [3], WBS has proven valuable for the study of the epidemiology of infectious diseases, such as Hepatitis E [4], Norovirus [5], and other enteric viruses [6]. However, WBS rose to prominence during the COVID-19 pandemic, when it proved valuable for the prevention and contention of outbreaks by detecting and quantifying viral genetic material using RT-PCR-based methods [7]. It must be noted that WBS data has proven to be significantly more useful when used in tandem with clinical reports, leading to robust risk assessment models that can be used to predict surges in cases, allowing for well-informed decision-making [8].

In WBS platforms, sampling usually happens at wastewater treatment plants (WWTPs), since municipal wastewater can be used as a representative sample of its served population, allowing for reduced bias in the resulting datasets [9]. Nevertheless, several studies have explored the potential of targeted systems to study the circulation of pathogens within specific, high-affluence areas, such as schools, hospitals, college campuses [10], and airports [11]. As discussed by Wolken et al. [12] strategic, targeted approaches are useful as a closer representation of the health status of subpopulations within a city and to prevent these buildings from becoming transmission hotspots, allowing for safer operations in the context of a pandemic. In previous studies, WBS has proven to be effective as an early warning system in the case of surges of COVID-19 cases, as sensitive and specific detection of viral genetic materials allowed for detection of both symptomatic and asymptomatic cases [13–17]. Moreover, longitudinal surveillance data can be used to reduce the intensity of individual clinical testing in periods when on-campus activities were returning to normalcy [18,19], and to evaluate the efficacy of mitigation measures, such as social distancing, compulsive facemask use [20], and vaccination [21], among others.

Previous efforts by our team explored the implementation of a WBS platform to survey the SARS-CoV-2 viral loads in the WWTPs of the Monterrey Metropolitan Area (MMA) in northern Mexico, correlating them with trends in clinical reports and urban mobility [22]. In our previous work, we implemented a similar platform to report the diversity and abundance of SARS-CoV-2 variants of concern in wastewater samples from the Mexico City’s sewage system [23]. We now report on the deployment and evaluation of a comprehensive WBS platform for the surveillance of SARS-CoV-2 on a building-by-building level across all the facilities of the largest private university in Mexico between January 2021 and June 2022. The institution operates 38 facilities in 19 of the 32 states of the country, serving 96,040 students as of 2022 [24].

This study’s objectives were to establish an extensive, building-by-building WBS platform across all facilities and to evaluate the correlation between the load of viral genetic materials in wastewater samples with epidemiologic reports. Finally, to evaluate the effectiveness of the mitigation measures taken by the institution, results from the central university campus, located in the MMA (Campus Monterrey from now on) will be explored and compared to data originating from wastewater samples from Dulces Nombres, the largest WWTP operating within the MMA.

## 2. Methods

Wastewater sampling across all university campuses in the study was coordinated by the Wastewater Surveillance Laboratory at Tecnológico de Monterrey (MARTEC) and the Instituto Tecnológico y de Estudios Superiores de Monterrey (Tec de Monterrey). As the project was a part of the strategies taken by the institution to tackle the COVID-19 pandemic and funding for operation was allocated internally, no further authorization was needed. Sampling at the Dulces Nombres WWTP took place after written authorization by Servicios de Agua y Drenaje de Monterrey, the public water utility operating within the MMA. All sampling procedures reported here complied with the guidelines established by the Norma Oficial Mexicana PROY-NMX-AA-003-SCFI-2019 (Secretaría de Comercio y Fomento Industrial, 2019).

### 2.1. Wastewater sampling protocols

**Fig 1:**
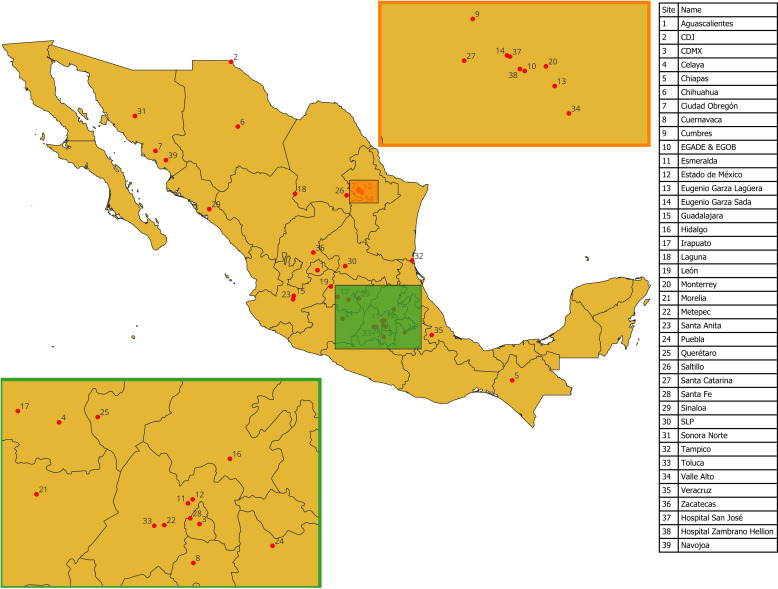
Geographical distribution of participating sampling sites across Mexico. Made with Natural Earth.

Wastewater sampling took place in 343 buildings across 39 participating facilities, including high schools, university campuses, and hospitals. The geographical distribution of campuses sampled in this study is shown in Fig 1. Samples were collected once a week between January 29, 2021 (denoted as week 5 from now on) and June 20, 2022 (denoted as week 78 from now on) in the wastewater discharge point of each participant building. Simple, 1 L grab samples were collected using high-density polyethylene (HDPE) bottles and stored at 4° C in ice packs for shipping to the central WBS laboratory, located within Campus Monterrey. In parallel, wastewater sampling was also conducted weekly at Dulces Nombres, the largest WWTP in the MMA, with a capacity of 7,500 liters per second and a served population of 1,695,589 inhabitants, in continuation of previous efforts by our team [22]. To limit the effect of daily variations in wastewater flow across all participant sampling sites (as no measures of wastewater flow could be collected for this study), sampling was conducted consistently on Mondays between 8 and 10 AM across all sampling sites.

### 2.2. Concentration and extraction of genetic materials

Immediately after reception, samples were pre-processed to concentrate viral genetic materials utilizing a PEG/NaCl precipitation method based on the one reported by Sapula et al. (2021) [25]. Briefly, 70 mL from each sample was collected in two, 50 mL Falcon tubes and clarified by centrifugation at 5,000 g for 5 minutes. Supernatants were collected into new tubes with a polyethylene glycol 8000 and NaCl solution, shaken manually until the phases were homogenized, and the genetic materials were precipitated by centrifugation at 12,000 g for 100 minutes. After discarding the liquid phase, pelleted genetic materials were resuspended in 300 µL of Milli-Q water and stored at −20 °C until extraction.

Extraction was conducted using a Water DNA/RNA Magnetic Bead Kit (IDEXX, Westbrook, Maine) adapted for automation using a KingFisher Flex instrument (Thermo Fisher, Waltham, Massachusetts) following the supplier’s guidelines. Final elution volumes were kept at 100 µL in all cases. RT-PCR detection of SARS-CoV-2 genetic materials was conducted on a QuantStudio 5 instrument (Applied Biosystems, Waltham, Massachusetts) using the SARS-CoV-2 RTPCR Test kit for wastewater samples (IDEXX, Westbrook, Maine). In accordance with the supplier’s guidelines, reactions consisted of 5 µL of SARS-CoV-2 mix, 5 µL of RNA Master Mix, and 5 µL of extracted genetic material. The RT-PCR program consisted of an initial hold for 15 min at 50 °C and then 1 min at 95 °C, followed by 45 amplification cycles of 95 °C for 15 s and 60 °C for 30 s. Ct values were collected for each sample into a database for analysis. A full list of all samples originating from college campuses in the study and the RT-PCR quantified viral loads is presented in S1 Table. Meanwhile, RT-PCR quantified viral loads found at Dulces Nombres WWTP during the study period are presented in S2 Table.

### 2.3. Epidemiological clinical data acquisition

**Fig 2:**
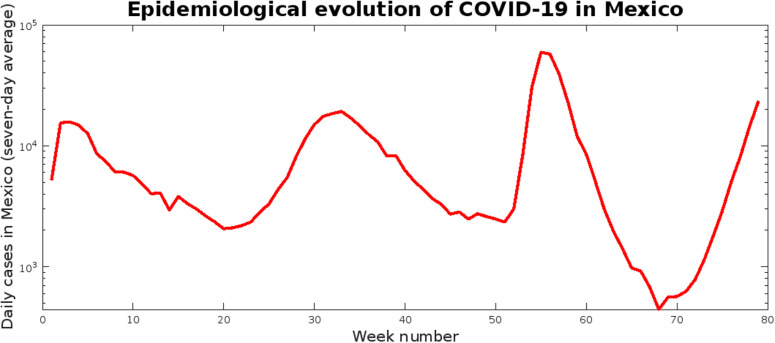
Weekly average of the daily new COVID-19 cases across Mexico during the study period (January 2021-June 2022).

Total daily reported COVID-19 cases by the state were obtained from the dashboard published by the National Council of Humanities, Sciences, and Technologies (CONAHCYT) with data provided by the General Direction of Epidemiology, a part of the Mexican Department of Health (available at https://datos.covid-19.conacyt.mx/). Here, the dataset corresponding to the study period is presented in S3 Table. For reference, a summary of the COVID-19 epidemiological trends across Mexico during the study period, presented as the average of the daily new cases reported by public health organizations each week, is presented in Fig 2.

Fluctuations in urban mobility in the MMA due to social distancing and other mitigation policies were accounted for using COVID-19 Community Mobility Reports published by Google in 2021 and 2022 for the Mexican state of Nuevo León (available at https://www.google.com/covid19/mobility/). Total changes in mobility were estimated by averaging the data from the six reported parameters (retail and recreation, groceries and pharmacies, parks, transit stations, workplaces, and residential). As data from wastewater surveillance was produced on a weekly basis, a weekly average was paired to each report. The dataset employed in the study is presented in S4 Table.

### 2.4. Data analysis and visualization

WBS and epidemiological data was originally captured and organized in spreadsheets using Microsoft Excel, saved as CSV files, and imported into Matlab R2024a for visualization and statistical analysis. First, buildings were studied individually by obtaining a timeseries for the RT-PCR quantified viral loads found weekly and calculating their correlation to clinical reports of COVID-19 incidence at the state level through Pearson correlation coefficients. Buildings where statistically significant correlations could be established (p-values below 0.05) and at least 20 weekly samples were taken during the study period were counted for each campus, and the building with the highest correlation coefficient was recorded. In buildings where less than 10 samples were collected, correlation coefficients and p-values were reported as NaN.

To limit the impact of inconsistencies during sampling due to restrictions at the sites or limited personnel, sites were clustered first by campus and by state for analysis. Four parameters were calculated weekly for each cluster: the average viral load of the weekly samples from each site (calculated as the sum of all the detected viral loads, expressed as copies per liter, divided by the number of samples taken from the site each week), the maximum viral load detected on the weekly samples from each site, the proportion of weekly positive samples (calculated as the number of positive samples from a site divided the total number of samples each week), and the proportion of buildings where the viral load was detected (calculated as the number of weekly positive samples divided by the total number of buildings sampled in the site). Pearson correlation coefficients and their associated p-values were calculated for the seven-day average daily new COVID-19 cases in the corresponding state and each of the four parameters discussed above to evaluate the concordance of results obtained from the detection of SARS-CoV-2 genetic materials in wastewater samples with the evolution of the pandemic in the populations around each of the campuses studied. Weeks in which no samples were collected and processed were not used for correlation analysis. All correlations with a p-value below 0.05 were considered statistically significant.

Finally, as the number of samples being processed by the laboratory varied significantly across the study period, a subsample of weekly data points was obtained and clustered analysis at the campus level was conducted again for this subset of data. In this subset, weekly reports where at least half of all the 343 buildings in the platform were sampled were selected.

### 2.5. Comparison between centralized and decentralized surveillance platforms in the Monterrey Metropolitan Area

For the state of Nuevo León, a comparative analysis between the data obtained from Campus Monterrey and Dulces Nombres was performed to evaluate the efficacy of the preventive measures taken by the institution. For this, Pearson correlation coefficients between the seven-day average new daily COVID-19 cases and the maximum viral load detected at Campus Monterrey and at Dulces Nombres, respectively, using both data from the entire study period and data obtained after urban mobility returned to pre-pandemic levels.

## 3. Results and discussion

### 3.1. Overview of wastewater sampling at Tecnológico de Monterrey

A total of 9,664 wastewater samples were collected, processed, and analyzed in this study. However, as seen in Table 1, the distribution of sampling sites across facilities was not homogeneous due to differences in area, the number of buildings and the size of the student population in each one. The campuses from where most samples were collected were Monterrey (1,395 samples), Querétaro (975), Guadalajara (634), Laguna (619), and León (551), all of them located in large metropolitan areas across Mexico. Similarly, the states from which more samples originated from were Nuevo León (2,006 samples), Mexico (987), Querétaro (975), Chihuahua (879), and Mexico City (855). As observed previously in similar efforts, such as the one reported by Wolken et al. (2023) [12], weekly sampling was not perfectly consistent due to factors including inaccessible sampling points, temporary closing of buildings due to surges in cases, lack of trained personnel, and scheduled holiday periods. As a result, while the total sampling period across the study extended for 74 weeks, the actual number of weeks in which sampling took place at Tecnológico de Monterrey facilities ranged from 62 at Campus Puebla to 14 at Hospital Zambrano. Likewise, the number of weeks in which sampling took place in at least one site in each state went from 62 in Puebla to 43 in Sinaloa. It must be noted that a total of two samples from Campus Veracruz were taken at the beginning of the study, but no further sampling was possible since the facility was closed shortly after. As such, it will not be considered in further data analysis.

**Table 1:** General summary of samples taken during the study, organized by campus and state of origin.

No.	Site	Total samples	Weekly samplings	Buildings
**Per campus**				
1	Monterrey	1,395	60	42
2	Querétaro	975	60	25
3	Guadalajara	634	60	28
4	Laguna	619	56	15
5	León	551	61	13
6	Chihuahua	502	60	14
7	Irapuato	378	59	10
8	CDJ	377	60	14
9	Estado de México	373	55	18
10	Zacatecas	337	51	8
11	Puebla	336	62	11
12	CDMX	281	60	8
13	Aguascalientes	258	61	9
14	Saltillo	236	52	7
15	Sonora Norte	228	57	10
16	Sinaloa	199	43	9
17	Toluca	194	52	8
18	SLP	158	56	6
19	Tampico	152	58	7
20	Santa Fe	140	57	9
21	Santa Catarina	139	47	4
22	Hidalgo	134	59	6
23	Cumbres	133	52	4
24	Morelia	112	58	7
25	Chiapas	98	59	4
26	Eugenio Garza Lagüera	90	45	3
27	Navojoa	88	54	5
28	Hospital Zambrano Hellion	70	14	5
29	Celaya	58	58	4
30	Ciudad Obregón	58	54	4
31	Cuernavaca	57	53	5
32	Esmeralda	56	53	4
33	Hospital San José	51	17	4
34	Valle Alto	50	44	4
35	Metepec	43	43	2
36	Eugenio Garza Sada	42	42	3
37	EGADE y EGOB	36	36	1
38	Santa Anita	24	24	2
39	Veracruz	2	2	1
**Per state**				
1	NUEVOLEON	2,006	61	59
2	MEXICO	987	61	23
3	QUERETARO	975	60	27
4	CHIHUAHUA	879	61	28
5	DISTRITOFEDERAL	855	57	21
6	HIDALGO	658	60	27
7	JALISCO	610	55	26
8	COAHUILA	477	61	20
9	SONORA	374	57	15
10	ZACATECAS	337	51	8
11	PUEBLA	336	62	11
12	AGUASCALIENTES	258	61	9
13	SINALOA	199	43	9
14	SANLUISPOTOSI	158	56	6
15	TAMAULIPAS	152	58	10
16	GUANAJUATO	134	59	6
17	MICHOACAN	112	58	7
18	CHIAPAS	98	59	5
19	MORELOS	57	53	5
20	VERACRUZ	2	2	1

**Fig 3:**
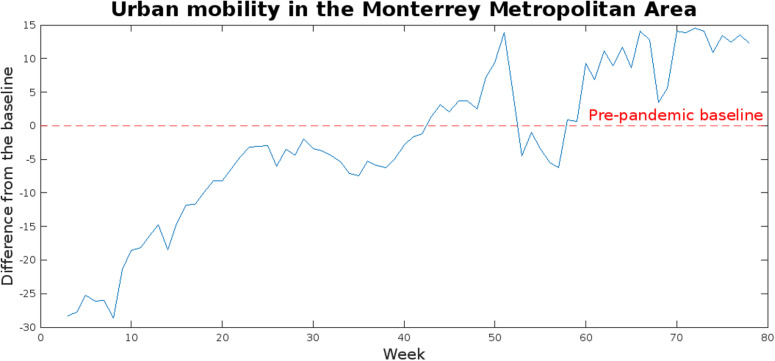
Changes in urban mobility in the MMA compared to the pre-pandemic baseline (January and February 2020), indicated with the dotted red line.

As seen in S1 Table, of the 9,664 samples from university campuses processed by the platform, SARS-CoV-2 genetic materials were successfully detected in 1658, resulting in an overall positivity rate of 17.16%. This is notoriously lower than the 62.71% positivity rate seen in the samples taken at Dulces Nombres WWTP (37 positives out of 59 total samples). However, in a study reporting on a similar decentralized, multi-site platform, Wolken et al. (2023) reported an overall positivity rate of 22.3% (486 positives out of 2176 samples), with a further 197 samples (9.1%) reported as inconclusive. Considering only the samples confidently reported as positive, the positivity rate in this study is 5.14% below the one reported by Wolken et al. (2023) [12]. It is likely that the reduced positivity rate in decentralized platform when compared to the centralized one is due to limited on-campus activities because of mobility restrictions, since those quarantining at home within the MMA would still be represented in the samples at Dulces Nombres but only those present at Campus Monterrey would be registered in the wastewater samples from the decentralized platform. Although data on campus occupancy could not be obtained for this study, COVID-19 Community Mobility Reports show noticeable decreases in urban mobility in the state of Nuevo Leon during 2021 (as presented in Fig 3), which could theoretically be used as a proxy of the levels of on-campus activity. Overall, this supports that decentralized wastewater surveillance platforms could benefit from a multi-site approach, and that clustered analysis can be effective for accurate decision-making.

### 3.2. Building-by-building evaluation of the surveillance platform

Correlation coefficients and associated p-values between the viral loads found in each sampling site across the surveillance platform and the weekly average of daily new COVID-19 cases in the state where the site was located is presented in S5 Table. Of the 343 sites in the study, 85 presented less than 10 registered samples during the study period and therefore no correlations could be confidently calculated. In the remaining 258 sites where sufficient sampling took place, statistically significant correlations between viral loads found in wastewater and clinical data could be established in 99 sites (38.37%).

As seen in Table 2, 31 of the 38 campuses in the study had at least one building where statistically significant correlations could be established. The campus with the highest number of representative buildings was Querétaro (15), followed by Monterrey (9) and León (5). On the other hand, no representative buildings were found in Cumbres, EGADE & EGOB, Eugenio Garza Sada, Hospital San José, Hospital Zambrano Hellion, Irapuato, Morelia, Navojoa, Santa Anita, Sinaloa, and Valle Alto. Interestingly, six of these eleven locations are high schools affiliated with the institutions, which are often smaller when compared to university campuses and could likely transition to remote activities more easily. Of the other five, one (EGADE & EGOB) is a specialized school within the MMA focusing on postgraduate studies in social sciences and business, and two (Morelia, Sinaloa) are among the smaller university campuses across the institution, as shown in the number of buildings reported in Table 1. As a result, it is likely that the on-campus populations in those locations, especially in periods where urban mobility was the lowest, was too small to be representative of their corresponding states (Nuevo Leon, Michoacán, and Sinaloa, respectively) Finally, no statistically significant correlations could be stablished for either of the two hospitals in the study, both of them in the MMA, likely as a result of insufficient sampling since sampling at these sites could only start at week 38 due to institutional restrictions.

**Table 2:** Overview of the building-by-building evaluation of the wastewater surveillance platform, reporting the number of representative buildings in each campus, the most representative building for each campus and its metrics.

Significant correlations	Best site	Site type	Total Samples	Positivity rate	Highest correlation	p value	Lowest cases detected
3	CEDIAM	Research laboratory	58	0.172	0.782	4.42E-13	21
2	Relajatec	Extracurricular activity site	22	0.046	0.971	6.95E-14	1655
3	CEDETEC (PTAR)	Wastewater collector	59	0.305	0.608	3.25E-07	171
2	Edificio aulas y oficinas	Mixed-used building	21	0.238	0.895	4.53E-08	427
1	Colector principal	Wastewater collector	55	0.091	0.529	3.29E-05	13
4	Prepa	Classrooms	41	0.146	0.667	1.88E-06	27
1	Colector principal	Wastewater collector	48	0.104	0.686	7.56E-08	149
1	Colector principal	Wastewater collector	45	0.133	0.492	5.99E-04	14
1	Colector principal	Wastewater collector	52	0.077	0.642	2.99E-07	171
3	Edificio 8	Classrooms	39	0.128	0.657	5.60E-06	674
2	Colector 2	Wastewater collector	45	0.089	0.576	3.53E-05	9
4	INGENIERIAS PRIMERA ETAPA	Classrooms	57	0.193	0.557	6.87E-06	31
1	carcamo ppal.	Wastewater collector	27	0.444	0.704	4.12E-05	5
3	Edificio III	Classrooms	42	0.143	0.616	1.43E-05	21
5	Colector PTAR	Wastewater collector	50	0.040	0.835	5.10E-14	508
1	Colector principal	Wastewater collector	40	0.075	0.801	5.46E-10	1246
9	Residencias 3	Student dormitory	59	0.153	0.890	4.22E-21	70
2	Residencias	Student dormitory	34	0.471	0.708	2.83E-06	12
15	Edif. 4 Profesional	Classrooms	59	0.186	0.813	5.10E-15	17
2	Auditorio	Mixed-used building	53	0.057	0.799	7.63E-13	29
1	Colector principal	Wastewater collector	36	0.167	0.729	4.71E-07	16
3	edificio 1	Classrooms	29	0.241	0.858	2.79E-09	171
3	Edificio 1	Classrooms	31	0.387	0.623	1.84E-04	14
4	Aulas IV	Classrooms	30	0.100	0.888	6.23E-11	113
1	Colector 1 (Aulas 1)	Wastewater collector	46	0.196	0.792	5.78E-11	38
2	AULAS 5	Classrooms	51	0.098	0.337	1.56E-02	167
2	LIFE	Extracurricular activity site	39	0.128	0.866	1.17E-12	152

Of the 27 campuses where correlations between clinical COVID-19 reports and SARS-CoV-2 viral loads in wastewater samples from at least one specific sampling site could be drown, wastewater collectors and classrooms were the most representative in 11 and 9 campuses, respectively. This is to be expected, as classrooms buildings often concentrated the most on-campus activities during the study period, as activities in other facilities, like cafeterias or gymnasiums were likely limited or suspended altogether. In the same line, samples taken at wastewater collection sites, whether connected to a local treatment facility or the municipal WWTPs, can be considered as composite samples representative of an entire facility. As a result, they are more likely to capture SARS-CoV-2 genetic materials consistently than sites in specific buildings, especially those where in-person activities were heavily restricted. In the remaining 8 campuses, the most representative sites were mixed-used buildings (2 instances), sites for extracurricular activities (2 instances), student dormitories (2 instances), and a research laboratory (1 instance).

Similarly to the trend seen in the overall platform, positivity rates varied significantly across the selected subset of representative buildings, from 47.1% at Residencias in Puebla to 4% at the wastewater collector in León. Consequently, due to the variations in positivity rates and the differences among the campuses and the cities they are located, the lowest number of daily new cases registered by public health authorities in weeks when a sample tested positive varied significantly among campuses, from 5 at Hidalgo to 1655 in CDJ. In any case, in 15 of these 27 campuses, positive samples were obtained in weeks when daily new COVID-19 case reports at the state level were below 100, and a further 7 campuses yielded positive samples when daily new COVID-19 clinical reports at the state level were between 100 and 200.

While these results support previous research indicating that decentralized surveillance platforms are capable of detection of SARS-CoV-2 even in settings were COVID-19 is remarkably low [26], the low positivity rate observed across the platform is an obstacle to correctly identifying the sensitivity of wastewater surveillance. Aside from issues arising from sample handling over long distances, as seen in the map presented in Fig 1, biomarker concentrations in wastewater samples have been reported to show significant variation, even when taken at sampling site and at similar times, because of degradation in sewage systems caused by temperature, pH, and exposure to light, among other factors, which have proven notoriously difficult to model with accuracy [27]. Additionally, nucleic acid extraction from wastewater samples often has highly variable yields; in a previous study by our team, we evaluated the performance of the RNA concentration and extraction procedures employed in this surveillance platform using a surrogate at known initial concentrations, and recovery rates varied between 35 and 67% [28]. All in all, these observations point at the viability of clustered analysis when evaluating decentralized, multi-site surveillance with highly variable positivity rates, as they can allow for more consistent data series and easier interpretability.

### 3.3. Sampling site clustering by campus and by state

**Fig 4:**
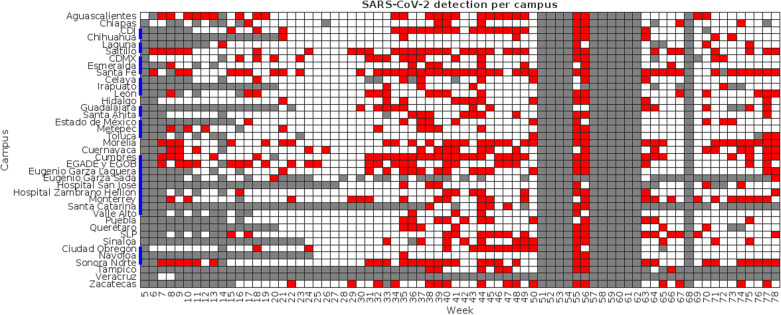
Summary of samplings across participant sites clustered by campus. The blue lines in the Y axis indicate groups of campuses within the same state.

**Fig 5:**
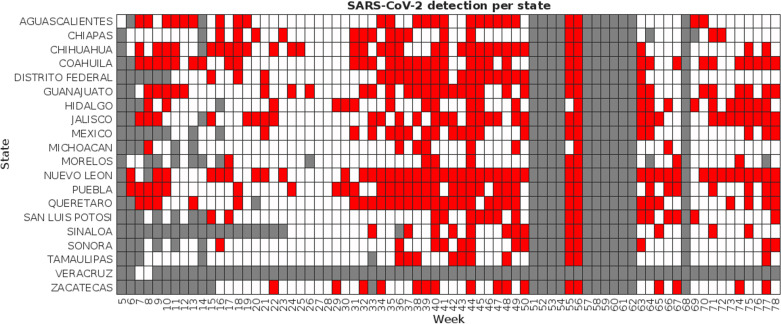
Summary of samplings across participant sites clustered by campus. The blue lines in the Y axis indicate groups of campuses within the same state.

Summarized sampling results when sapling sites were clustered by campus are presented in Fig 4, while Fig 5 presents the results when sites were clustered by state. The blue lines in Fig 4 show groups of campuses that are in the same state. Red squares show weeks when sampling was conducted at a given site and at least one of the sampled buildings tested positive for SARS-CoV-2 genetic materials, white squares show weeks when sampling was conducted but no viral loads were detected, and grey squares show weeks when no sampling was conducted.

As shown in Table 3, the maximum viral load and the average viral load in the weekly samples taken in each site showed the most consistent correlation to the seven-day average of new daily COVID-19 cases in the corresponding state, both when campuses were analyzed individually (average correlation coefficients of 0.448 ± 0.302 and 0.427 ± 0.303, respectively) and when campuses were clustered by state (0.465 ± 0.276 and 0.438 ± 0.277). The correlation with the proportion of positive samples and positive buildings fell significantly (0.296 ± 0.197 and 0.309 ± 0.201 for individual campuses, 0.307 ± 0.153 and 0.317 ± 0.180 for states), likely due to the inconsistencies in samples discussed previously, which led to significant variations in the number of samples being obtained each week. The number of sites where correlation proved statistically significant (p-values below 0.05) showed similar behavior: correlation to the maximum viral load per site showed to be significant in 25 of the 38 campuses and 13 of the 19 states, followed by the average viral load per campus or state (24/38 and 13/19, respectively).

**Table 3:** Average correlation coefficients for the average viral load, maximum viral load, proportion of positive samples, and proportion of positive buildings across sampling sites, by campus and by state.

	Correlation with seven-day average new daily cases			
	Average viral load	Maximum viral load	Proportion of positive samples	Proportion of positive buildings
**Per campus**				
Average correlation coefficient (Pearson)	0.427 ± 0.303	0.448 ± 0.302	0.296 ± 0.197	0.309 ± 0.201
Sites with statistically significant correlation	24	25	22	22
Percentage of sites with statistically significant correlation	63.16%	65.79%	57.89%	57.89%
**Per state**				
Average correlation coefficient (Pearson)	0.438 ± 0.277	0.465 ± 0.276	0.307 ± 0.153	0.317 ± 0.180
Sites with statistically significant correlation	13	13	12	11
Percentage of sites with statistically significant correlation	68.42%	68.42%	63.16%	57.89%

As suggested previously, correlation coefficients vary greatly among campuses, going from 0.9004 at Hospital San José to −0.0942 in Campus Sinaloa, although most campuses show positive correlation coefficients and 24 out of 38 show coefficients above 0.4. The complete list is presented in Table 4 of number of weekly samplings and the average coverage rate (calculated as the average of the ratios between samples taken on each campus per week and the total number of sampling sites within the campus) for each campus, the Pearson correlation between the maximum viral load found weekly on each campus and the seven-day average of new daily COVID-19 cases in the state where the campus is located, and its related p-value.

**Table 4:** Correlation coefficients between the maximum viral load and the seven-day average of new daily COVID-19 cases across campuses.

Campus	Weekly samplings	Coverage rate	Correlation coefficient (Pearson)	p-value
Hospital San José	17	0.750 ± 0.000	0.900	8.383E-07
Celaya	58	0.250 ± 0.000	0.843	1.073E-16
Zacatecas	51	0.826 ± 0.216	0.828	6.592E-14
Querétaro	60	0.650 ± 0.339	0.818	1.435E-15
Metepec	43	0.500 ± 0.000	0.798	1.409E-10
Hospital Zambrano Hellion	14	1.000 ± 0.000	0.751	1.978E-03
Santa Catarina	47	0.739 ± 0.072	0.707	2.776E-08
León	61	0.695 ± 0.324	0.692	6.501E-10
Ciudad Obregón	54	0.269 ± 0.094	0.681	1.444E-08
Estado de México	55	0.377 ± 0.13	0.642	1.240E-07
Puebla	62	0.493 ± 0.334	0.640	2.155E-08
Esmeralda	53	0.264 ± 0.102	0.631	4.035E-07
Sonora Norte	57	0.400 ± 0.206	0.616	3.371E-07
Hidalgo	59	0.379 ± 0.247	0.603	4.244E-07
CDMX	60	0.585 ± 0.193	0.602	3.681E-07
Cuernavaca	53	0.215 ± 0.066	0.601	1.948E-06
Santa Fe	57	0.273 ± 0.075	0.598	9.178E-07
SLP	56	0.470 ± 0.232	0.595	1.334E-06
Laguna	56	0.737 ± 0.262	0.589	1.828E-06
Guadalajara	60	0.377 ± 0.174	0.575	1.574E-06
Eugenio Garza Lagüera	45	0.667 ± 0.000	0.574	3.701E-05
Irapuato	59	0.641 ± 0.243	0.574	1.989E-06
Saltillo	52	0.648 ± 0.195	0.527	6.084E-05
Monterrey	60	0.554 ± 0.341	0.486	8.288E-05
Toluca	52	0.466 ± 0.277	0.328	0.018
Aguascalientes	61	0.470 ± 0.187	0.247	0.055
Chiapas	59	0.415 ± 0.157	0.230	0.079
Valle Alto	44	0.284 ± 0.126	0.198	0.198
Tampico	58	0.374 ± 0.209	0.175	0.189
Navojoa	54	0.326 ± 0.097	0.084	0.544
Santa Anita	24	0.500 ± 0.000	0.061	0.777
CDJ	60	0.449 ± 0.312	0.059	0.652
Cumbres	52	0.639 ± 0.205	0.016	0.911
Morelia	58	0.276 ± 0.140	−0.007	0.960
Chihuahua	60	0.598 ± 0.149	−0.027	0.836
EGADE y EGOB	36	1.000 ± 0.000	−0.030	0.864
Eugenio Garza Sada	42	0.333 ± 0.000	−0.091	0.569
Sinaloa	43	0.514 ± 0.247	−0.094	0.548

Just as observed when clustering sampling sites by campus, correlation coefficients vary greatly among states, going from 0.8280 in Zacatecas to −0.0942 in Sinaloa, although most states show positive correlation coefficients, and 13 out of 19 show coefficients above 0.4. The complete list is presented in Table 5 with the number of weekly samplings and the average coverage rate (calculated as the average of the ratios between samples taken on each state per week and the total number of sampling sites within the campus) for each state, the Pearson correlation between the maximum viral load found weekly on each state and its seven-day average of new daily COVID-19 cases, and the related p-value.

**Table 5:** Correlation coefficients between the maximum viral load and the seven-day average of new daily COVID-19 cases across states.

State	Weekly samplings	Coverage rate	Correlation coefficient (Pearson)	p-value
ZACATECAS	51	0.826 ± 0.216	0.828	6.592E-14
QUERETARO	60	0.650 ± 0.339	0.818	1.435E-15
GUANAJUATO	61	0.952 ± 0.398	0.657	8.834E-09
MEXICO	55	0.616 ± 0.255	0.643	1.208E-07
PUEBLA	62	0.493 ± 0.334	0.640	2.155E-08
SONORA	57	0.596 ± 0.232	0.635	1.143E-07
COAHUILA	61	0.711 ± 0.239	0.632	4.829E-08
HIDALGO	59	0.379 ± 0.247	0.603	4.244E-07
MORELOS	53	0.215 ± 0.066	0.601	1.948E-06
SAN LUIS POTOSI	56	0.470 ± 0.232	0.595	1.334E-06
DISTRITO FEDERAL	57	0.882 ± 0.306	0.589	1.445E-06
JALISCO	60	0.392 ± 0.171	0.575	1.575E-06
NUEVO LEON	61	0.747 ± 0.451	0.486	7.067E-05
AGUASCALIENTES	61	0.47 ± 0.187	0.247	0.055
CHIAPAS	59	0.415 ± 0.157	0.230	0.079
TAMAULIPAS	58	0.374 ± 0.209	0.175	0.189
MICHOACAN	58	0.276 ± 0.14	−0.007	0.960
CHIHUAHUA	61	1.029 ± 0.432	−0.018	0.889
SINALOA	43	0.514 ± 0.247	−0.094	0.548

While satisfactory correlation between RT-PCR quantified SARS-CoV-2 viral loads found in wastewater samples across the sampling sites in the study and the clinical reports on COVID-19 incidence could be stablished for most university campuses integrated into the surveillance platform, sample positivity rate was notoriously influenced by the introduction of measures to limit the transmission of SARS-CoV-2 during on-campus activities. These included mandatory facemask-wearing, vaccination, and reduced levels of in-person activities were implemented across the entire period of study, as such measures have proven to intervene in viral transmission pathways within buildings, as studied by Tsang et al. (2023) [29]. In fact, data from the surveillance platform was used to inform institutional authorities in support of such preventative measure in a mechanism like the one reported by Wang et al. (2022) [16]. Similar observations were made in a study where surveillance took place at both the University of California at Davis and the entire city of Davis, California, as the level in which these preventive measures were implemented varied significantly during the duration of the pandemic [30]. As such, a Bayesian sequential analysis was used to adequately correlate viral loads in wastewater and the test positivity rate, used as a measure of COVID-19 incidence, to enhance surveillance as containment measures progressively eased [30]. Additionally, data from decentralized platforms, like the one presented here, could be used in tandem with centralized platforms to better support strategies for a safe return to in-campus activities while minimizing pathogen transmission.

However, estimating the efficacy of outbreak prevention measures using WBS data has proven difficult, as adequately modeling the rates of viral shedding [31] and RNA degradation in sewage systems [27] has been identified as a challenge. Moreover, reports on the implementation of extensive, targeted surveillance platforms, such as the ones by Bowes et al. (2023) [32] and Wolken et al. (2023) [12], have stressed the importance of adequate standardization of sampling, pretreatment, concentration and extraction procedures, as well as the proper identification the sensitivity, specificity and limit of detection of the technique used for SARS-CoV-2 viral load detection to minimize systematic variation and obtain complete, robust data sets.

### 3.4. Study of a subset of datapoints based on sampling rate

**Fig 6:**
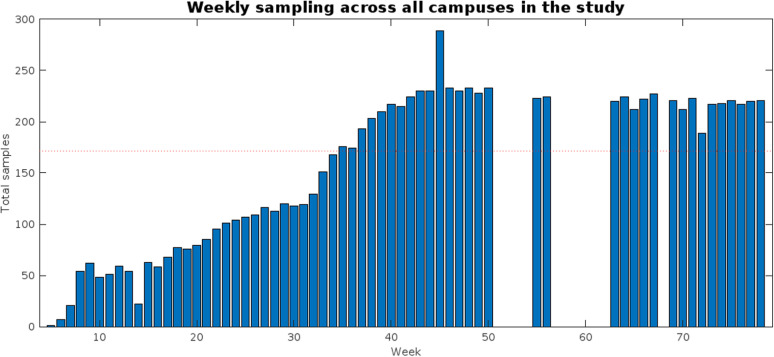
Number of total samples processed each week across all campuses in the surveillance platform. The dotted red line represents a number of samples equal to half the total buildings registered in the study.

An overview of the total number of samples obtained each week throughout the whole surveillance platform is presented in Fig 6. Consistent with the observations made in the previous section, sampling across sites was not consistent throughout the entire study period. In fact, sampling did not start at the same time for all locations; instead, sites were progressively added to the platform during the first half of the study period in accordance with the needs of the institution and the capacity of the laboratory. The number of samples being processed weekly stabilized after week 35, when at least half of all the sites registered in the study were being consistently sampled each week. This is a particularly relevant consideration to be made during analysis, as insufficient sampling may have limited the ability of the laboratory to adequately detect surges in COVID-19 reports, as seen during weeks 20–30 (May-July 2021). Therefore, correlations at the campus levels were recalculated at the campus level using samples from week 35 onwards to reduce the impact of the inconsistent sampling rate in the correlations observed.

The subsample obtained from week 35 onwards contains a total of 7,229 samples, 74.8% of all samples collected across the study period. Of these, SARS-CoV-2 genetic materials were successfully detected in 1435, yielding a positive rate of 19.85%, slightly higher than the 17.16% found across the entire study period and closer to the 22.3% reported by Wolken et al. (2023) [12]. As presented in Table 6, 27 of the 38 campuses in the study showed statistically significant correlations between the highest viral load found across all samples taken and the reported COVID-19 incidence in the same week between weeks 35 and 78, compared to the 25 found when using the data across the entire study period. The average correlation coefficient found across campuses during the selected time window was 0.508 ± 0.325, compared to the 0.448 ± 0.302 obtained when using the entire database. While none of the previously established correlations were lost when using the subsample, two more statistically significant correlations (Chiapas and Chihuahua) could be established.

**Table 6:** Correlation coefficients between the maximum viral load and the seven-day average of new daily COVID-19 cases across campuses in weeks 35 to 78.

Campus	Weekly samplings	Detection weeks	Correlation	p-value
Hospital San José	17	8	0.9004	8.38E-07
Metepec	33	3	0.8826	1.10E-11
Celaya	33	6	0.8662	7.40E-11
Querétaro	33	23	0.8660	7.62E-11
Zacatecas	33	13	0.8387	1.10E-09
Chiapas	31	9	0.7720	3.65E-07
Chihuahua	33	12	0.7662	2.03E-07
Puebla	33	22	0.7634	2.38E-07
Santa Anita	33	7	0.7572	3.39E-07
Hospital Zambrano Hellion	14	6	0.7507	0.00198
Cuernavaca	31	7	0.7256	3.86E-06
Ciudad Obregón	32	5	0.7213	3.20E-06
Estado de México	33	13	0.7108	3.56E-06
León	33	10	0.7005	5.66E-06
Hidalgo	33	17	0.6840	1.14E-05
Esmeralda	31	3	0.6807	2.51E-05
CDMX	33	22	0.6511	4.07E-05
Sonora Norte	33	9	0.6501	4.23E-05
Santa Catarina	33	11	0.6462	4.86E-05
Guadalajara	33	24	0.6272	9.38E-05
Eugenio Garza Lagüera	31	8	0.6254	0.0002
Sinaloa	33	15	0.6091	0.0002
Irapuato	33	15	0.6058	0.0002
Laguna	32	17	0.5899	0.0004
SLP	31	6	0.5673	0.0009
Monterrey	32	29	0.4928	0.0042
Toluca	33	12	0.3723	0.0329
Saltillo	10	1	0.2711	0.4486
Aguascalientes	33	15	0.2549	0.1523
Tampico	33	10	0.1732	0.3351
Navojoa	33	4	0.0856	0.6359
CDJ	32	10	0.0324	0.8606
Cumbres	33	9	0.0124	0.9453
Morelia	33	7	0.0013	0.9943
EGADE y EGOB	29	6	−0.0164	0.9327
Santa Fe	32	12	−0.0782	0.6706
Eugenio Garza Sada	28	3	−0.1057	0.5924
Valle Alto	31	8	−0.134	0.4725

While the performance of the building-by-building WBS platform was satisfactory, it is important to highlight the challenges related to the operation of such a large network of sampling sites distributed in a vast geographical area, including incomplete sample collection due to unforeseeable circumstances during the study period and inadequate sampling handling during transportation, likely a result of insufficient cold chains, as reviewed by Bengiovanni et al. (2020)[33]. These obstacles led to the observed variation in the number of weekly samplings taken per campus and by state, in the effective coverage rates, and the representativity obtained in each campus when compared to the epidemiological data reported by the public health authorities. Extensive networks would be ideal to obtain more detailed information that could be used for better risk assessment models, especially in areas that are particularly prone to disease outbreaks [10]; however, limited datasets are to be expected as surveillance platforms expand, especially in Low-to-Middle Income Countries like Mexico, where such systems are often operated with limited resources [34,35]. Similar surveillance efforts conducted in the future would likely benefit from a more targeted approach, where sampling sites are set strategically, to allow for more sustained, consistent coverage while retaining sample representativity. A strategy to identify sampling sites that can be highly representative of the population in the corresponding sewageshed, accounting for both population size and socioeconomical factors such as social vulnerability, has been developed by Daza–Torres et al. (2024) [36].

In cases of public health emergencies where extensive, sustained surveillance is crucial for public health, as in the case of the COVID-19 pandemic, it is likely that WBS platforms would be more effective when operated collaboratively across institutions as long as the procedures for sample handling, pretreatment, and viral genetic material extraction and detection are properly standardized and proven reproducible, as observed by Oyervides-Muñoz et al. (2024) [28].

### 3.5. Comparisons between data from a college campus and a WWTP

**Fig 7:**
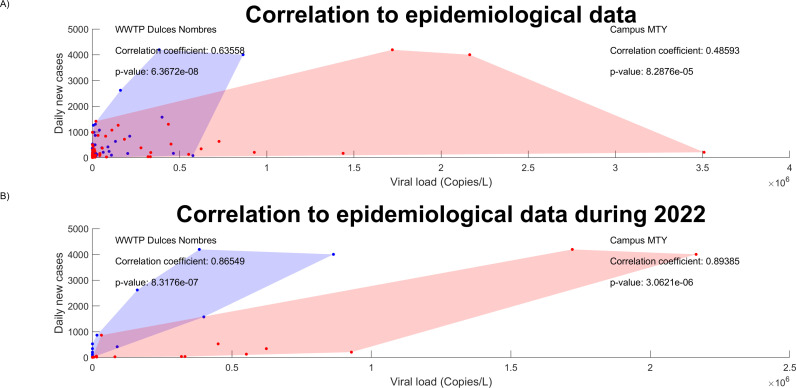
Correlation between the seven-day average of daily new cases of COVID-19 in the state of Nuevo León and the viral load detected at Campus Monterrey (blue) and Dulces Nombres (red). A) Including the entire period of study. B) Only considering data from January to June 2022.

To offer insights on the effectiveness of the epidemiological contention and prevention taken by Tecnológico de Monterrey (including reduced in-person activities, compulsive mask wearing and vaccination), data obtained from wastewater surveillance in Campus Monterrey and the Dulces Nombres WWTP, both located in the MMA, was compared to epidemiological data reported by public health authorities for the state of Nuevo León. After eliminating weeks when no sampling took place, correlation between the maximum viral load found on all the samples originating from Campus Monterrey each week and the corresponding seven-day average of new daily cases of COVID-19 reported in Nuevo León was 0.486 (n: 60, p-value: 8.2876x10^−5^). For the viral load detected at Dulces Nombres, the observed correlation coefficient reached 0.6356 (n: 59, p-value: 6.3672^−8^). Plots presenting the distribution of the data from each weekly sampling is presented in Fig 7A.

Since samples from both sites originate from the same metropolitan area and were transported a similar distance to the central laboratory following standard practices for genetic material conservation, it can be suggested that the difference in their degree of correlation to the epidemiological situation in the state is due to the different populational dynamics they represent. Campus Monterrey, like most higher education facilities in Mexico, greatly limited its in-person activities during the pandemic; maintenance personnel, researchers, postgraduate students, and security guards were still going regularly into the facilities, but most of the undergraduate students, faculty, and administrative personnel conducted their activities remotely. Inversely, Dulces Nombres is the biggest collection point for domestic and industrial wastewater in the MMA and, as a result, is representative of the entire population within its catchment area, regardless of whether they adhered to social distancing regulations, presented any symptoms of COVID-19, or received a clinical test and were reported to the epidemiological databased compiled and published by public health authorities. Therefore, it can be observed that, while extensive wastewater surveillance at Campus Monterrey was useful for a safe continuation of strategic activities at campus that could not be conducted remotely, WBS platforms centered on WWTPs were still more representative of the epidemiological situation in the MMA, where the implementation of transmission measure prevention doesn’t impact in wastewater viral load, contrary to what has been observed on the campuses.

As previously shown in Fig 3, mobility surpassed the pre-pandemic baseline in week 43 (the last week of October 2021) and remained above the baseline throughout 2022 (week 52 onwards) with the exceptions of weeks 53–57 (in January 2022), likely due to the holiday season and increased cases in the winter. This indicates a relative return to normalcy in the populational dynamics of the state. Taking this into account, correlations between the maximum viral load found each week at Campus Monterrey and at Dulces Nombres with the seven-day average of new daily cases in the state of Nuevo Leon between January and June of 2022. As expected, correlation increased noticeably in both cases due to less stringent epidemiological containment measures, reaching correlation coefficients of 0.8655 (n: 20, p-value: 8.3176x10^−7^) for Dulces Nombres, and 0.8938 (n: 16, p-value: 3.0621x10^−6^) for Campus Monterrey, as presented in Fig 7B. Moreover, the fact that correlation coefficients for both sites were so close after week 52 indicates that the dynamics in campus occupation at Campus Monterrey returned to normal progressively between late 2021 and early 2022, in tandem with the reestablishment of pre-pandemic urban mobility patterns in the MMA. While this may hint at the effectivity of the measures taken at Campus Monterrey to limit SARS-CoV-2 transmission throughout 2021, which were implemented in conjunction with the rest of the facilities of Tecnológico de Monterrey across Mexico, it must be noted that routinary surveillance at Campus Monterrey stopped after June 2022 due to a cut in funding by the institution; a more detailed analysis of the epidemiological dynamics in the institution after on-campus activities returned to normal levels would require longer surveillance.

## 4. Limitations and perspectives

This work offers important insights into the operation of a large WBS platform for the surveillance of SARS-CoV-2 across 39 facilities across Tecnológico de Monterrey, encompassing high schools, college campuses, and hospitals in 20 of the 32 federal entities of Mexico. The performance of such a platform was evaluated through comparisons to epidemiological data reported by public health authorities and, in the case of Campus Monterrey (the largest of all participant facilities), to data obtained from samples taken at Dulces Nombres, the largest WWTP in the MMA. While it was observed that viral loads detected in wastewater samples were correlated with the amount of daily new COVID-19 cases in 25 campuses across 13 states, and that WBS could be used as an effective strategy to support epidemiological contention measures, as shown for the case of Campus Monterrey, opportunities for improvement of WBS platforms persist.

However, the main limitation of this study is that, while SARS-CoV-2 viral load data could be successfully compiled across all locations in the platform, no data on campus occupancy, wastewater flow, or results of COVID-19 testing on each campus could be obtained for analysis in this study, limiting the analysis of how changes in on-campus activities could have impacted the correlation between viral loads found in wastewater samples from university campuses and clinical reports of COVID-19 incidence. This was because the wastewater surveillance platform reported here was operated as an independent research project by our research team and its involvement with the institutional authorities following the evolution of the pandemic across all campuses was limited. Implementation of similar surveillance platforms would greatly benefit from closer colaboration with public health authorities and the healthcare sector, providing a more comprehensive database that allows for efficient preventive and containment efforts as in-person activities are resumes during sanitary emergencies similar to COVID-19.

Sampling across a large network of sites across participating facilities was relevant for the institution, as it offered data to guide effective preventive efforts for a safe continuation of priority in-person activities. However, encompassing such a large set of sampling points into a single platform operated from a single laboratory made consistent coverage across campuses (defined here as the rate of total weekly samples from a facility over the total amount of participant buildings in the facility) difficult. A notable example is the limited number of samples collected at the two hospitals (Hospital San José and Hospital Zambrano Hellion), operated by the School of Medicine of the institution: as both were enabled to receive hospitalized COVID-19 patients, routine sampling could only be established in week 38 and could not be sustained after week 56, since the Omicron-related surged in cases brought the Mexican healthcare system under substantial strain [23]. Such considerations are likely to be important for the operation of similar surveillance platforms during future pandemic outbreaks, especially in low-to-middle-income countries.

While taking only the weekly maximum viral load for each campus for correlation studies helped with consistency when handling such an incomplete database, as shown by the performance metrics reported in the previous section, significant variance in coverage rates may have contributed to limited correlation between WBS data and epidemiological reports when compared to surveillance efforts centered at WWTPs. Future efforts towards sustained, extensive surveillance platforms, either for detection of endemic pathogens such as Influenza or Norovirus, or future epidemiological outbreaks akin to SARS-CoV-2, may benefit from a more strategic selection of sampling points that can be studied consistently and yield results that can be representative of general populational dynamics, as previously suggested by Safford et al. (2022) [37]. However, while detection of SARS-CoV-2 genetic materials in wastewater samples from a specific building was used by the institution to regulate campus occupancy to reduce transmission within the campuses, the data presented here is insufficient to discuss the effectiveness of the measures as data on campus occupancy could not be obtained and the amount of samples taken after urban mobility levels had returned to normal were limited. Further studies to assess the efficacy of measures to limit the transmission of COVID-19 and similar viral pathogens in university settings, including limited in-person activities, facemask use, or vaccination rates, among others, are still needed.

Moreover, standardized, reproducible sample processing methods will be crucial for any WBS platform, as inconsistent viral load detection and quantification may hinder data comparability and analysis, as discussed previously by Bowes et al. (2023) [32]. Moreover, levels of biomarkers in wastewater have been observed to vary significantly because of changes in the flow at the designated sampling site, fluctuations in human activity, and possible degradation of the molecule of interest [38]. While the surveillance effort reported here relied on simple grab samples, a limitation that could be mitigated by clustering different sites by campus and by state during data analysis, smaller, more targeted platforms could benefit from the use of composite sampling or implementation of passive sampling technologies, although their applicability for wastewater surveillance of pathogens still requires further studies [39]. Another crucial aspect of this platform is that all samples were analyzed at a central laboratory in the Monterrey Metropolitan Area, to which all samples were shipped. As seen on the map presented in Fig 1, the distance between sampling sites and the laboratory varied significantly, so adequate sample handling procedures had to be implemented. While the impact of sample transportation on the integrity of the biomarkers it contains is well studied [33], studies regarding how potential disruptions in cold chains during sample transportation can influence the results of wastewater surveillance platforms and how to account for this during data analysis still need to be conducted.

Finally, integration of viral load quantification in wastewater samples into complete epidemiological models that could be used for risk assessment and prevention during a public health emergency, such as the one posed by SARS-CoV-2, remains as an area of opportunity for WBS. While certain directions for the interpretation of WBS data as an indicator of public health status have been discussed elsewhere [8], data normalization remains a challenge as no universal wastewater biomarker for population size and levels of human activity have been agreed upon, although several options have been suggested [40]. In any case, WBS should not be thought of as a substitute for individual clinical tests, but as a complement for more robust epidemiological data. This work focuses only on the main outcomes of the WBS platform deployed at Tecnológico de Monterrey, while their integration into epidemiological models will be explored in future studies.

## 5. Conclusions

Overall, the extensive, building-by-building WBS platform deployed across Tecnológico de Monterrey successfully reflected the evolution of the COVID-19 epidemic in 25 of the 38 facilities in the study and provided valuable insights for effective epidemiological containment during 2021, allowing for the continuation of priority in-person activities. Moreover, implementing a WBS platform targeting educative institutions like university campuses proved a suitable proxy to study the epidemiological dynamics in tandem with clinical reports published by public health authorities in larger communities, like states.

Additionally, this study evaluates the optimal strategy to obtain correlations between viral loads in wastewater samples from individual buildings and published clinical reports, proving that clustering sampling points by campus or state and using the maximum viral load found in each cluster is a feasible strategy to generate robust, insightful datasets for informed decision-making. In fact, data from Campus Monterrey became more representative of the public health condition in the state of Nuevo León after urban mobility went back to pre-pandemic levels at the start of 2022, indicating that the altered populational dynamics within the campus during 2021 due to limited in-person activities were effective to prevent COVID-19 outbreaks within the facility.

However, further studies regarding the optimization of such a vast platform and the integration of WBS data into broader epidemiological models are still needed for the development of robust, efficient surveillance platforms for future public health emergencies.

## Supporting information

S1 Table Full list of all samples collected and analyzed by the surveillance platform. (XLSX)

S2 Table Viral loads found at Dulces Nombres. (XLSX)

S3 Table Daily new COVID-19 cases reported in each state of Mexico. (XLSX)

S4 Table Urban mobility reports during the study period in Nuevo Leon. (XLSX)

S5 Table Building-by-building evaluation of the surveillance platform. (XLSX)
