# Correction: Academic institution extensive, building-by-building wastewater-based surveillance platform for SARS-CoV-2 monitoring, clinical data correlation, and potential national proxy

**DOI:** 10.1371/journal.pgph.0005250

**Published:** 2025-09-24

**Authors:** Arnoldo Armenta-Castro, Mariel Araceli Oyervides-Muñoz, Alberto Aguayo-Acosta, Sofia Liliana Lucero-Saucedo, Alejandro Robles-Zamora, Kassandra O. Rodriguez-Aguillón, Antonio Ovalle-Carcaño, Roberto Parra-Saldívar, Juan Eduardo Sosa-Hernández

The Funding statement for this article is incorrect. The correct Funding statement is as follows: This research was funded by the Fundación FEMSA project entitled “Unidad de respuesta rápida al monitoreo de COVID19 por agua residual” (grant number NA to RPS). This work was supported by Tecnologico de Monterrey through the project Challenge-Based Research Funding Program 2022 (Muestreador Pasivo I026 - IAMSM005 - C4-T1 – T to JESH). Research time for R Parra Saldivar is funded by Research England, UKRI. The funders had no role in study design, data collection and analysis, decision to publish, or preparation of the manuscript.
